# Secondary Hemophagocytic Lymphohistiocytosis Triggered by Visceral Leishmaniasis Due to *Leishmania infantum*: A Case Report

**DOI:** 10.3390/reports9030268

**Published:** 2026-08-12

**Authors:** Christina Velliou, Anna Varouktsi, Iraklis Leonidis, Anastasia Sarvani, Nikoleta Moutsou, Theocharis Koufakis, Dimitrios Patoulias

**Affiliations:** Second Propedeutic Department of Internal Medicine, Hippokration General Hospital, Aristotle University of Thessaloniki, 54642 Thessaloniki, Greece; christinevelliou@gmail.com (C.V.); annavarouktsi@gmail.com (A.V.); leonidismd@gmail.com (I.L.); natasasar91@gmail.com (A.S.); nikoletamoutsou@gmail.com (N.M.); thkoyfak@auth.gr (T.K.)

**Keywords:** hemophagocytic lymphohistiocytosis, visceral leishmaniasis, *Leishmania infantum*, hyperferritinemia, cytokine storm

## Abstract

**Background and Clinical Significance:** Hemophagocytic lymphohistiocytosis (HLH) is a life-threatening hyperinflammatory syndrome characterized by uncontrolled activation of macrophages and cytotoxic lymphocytes. Secondary HLH is most commonly associated with infections, malignancies, and autoimmune disorders. Visceral leishmaniasis (VL) is an uncommon infectious trigger of HLH, and the considerable overlap in clinical manifestations frequently delays diagnosis. Early recognition of the infectious trigger is essential, because prompt targeted therapy may prevent the need for prolonged immunosuppressive treatment and substantially improve outcomes. **Case Presentation:** A 68-year-old livestock farmer from northern Greece presented with a one-month history of persistent fever, fatigue, and night sweats. Laboratory evaluation demonstrated pancytopenia, severe hyperferritinemia, hypertriglyceridemia, and elevated soluble interleukin-2 receptor (sCD25) levels. After extensive, relevant screening for other underlying etiologies, which proved to be negative, bone marrow examination revealed hemophagocytosis, while anti-*Leishmania* serology and polymerase chain reaction (PCR) analysis of bone marrow aspirate confirmed infection with *Leishmania infantum*. Treatment with liposomal amphotericin B along with intravenous dexamethasone resulted in rapid clinical and laboratory improvement. Serial ferritin and sCD25 measurements closely paralleled clinical recovery, supporting their potential usefulness as biomarkers of treatment response. The patient remained asymptomatic at one-month follow-up. **Conclusions:** Secondary HLH associated with VL is rare, but potentially fatal. Clinicians should maintain a high index of suspicion in patients presenting with prolonged fever, splenomegaly, cytopenia, and marked hyperferritinemia, particularly in endemic regions. Early diagnosis and prompt initiation of targeted therapy are associated with favorable outcomes.

## 1. Introduction and Clinical Significance

Hemophagocytic lymphohistiocytosis (HLH) is a severe hyperinflammatory syndrome characterized by uncontrolled activation of macrophages, cytotoxic T lymphocytes, and natural killer cells, resulting in excessive cytokine release and potentially fatal multiorgan dysfunction [1,2,3,4,5]. HLH is classified into primary (familial) and secondary (acquired) forms. Secondary HLH occurs predominantly in adults and is most commonly associated with infections, malignancies, autoimmune diseases, and, less frequently, administration of immunomodulatory therapies [2,3,4].

Visceral leishmaniasis (VL), also known as kala-azar, is a systemic parasitic disease caused by protozoa of the genus *Leishmania*. In the Mediterranean region, including Greece, *Leishmania infantum* is the predominant causative species [6,7,8]. The parasite is transmitted by infected female phlebotomine sandflies and primarily affects the reticuloendothelial system, typically presenting with prolonged fever, splenomegaly, weight loss, and pancytopenia, while, if left untreated, it may be fatal [6,7,8].

Although relatively uncommon, VL is a well-recognized infectious trigger of secondary HLH [9,10]. The coexistence of these conditions presents a significant diagnostic challenge, because both disorders share similar clinical and laboratory manifestations, including prolonged fever, splenomegaly, cytopenias, hypertriglyceridemia, and marked hyperferritinemia [9,10]. Consequently, diagnosis may be delayed, increasing the risk of progressive hyperinflammation, multiorgan dysfunction, and finally death [3,4].

We report the case of a 68-year-old immunocompetent man with secondary HLH triggered by *Leishmania infantum* infection. The objective of this report is to describe a case of secondary HLH triggered by *Leishmania infantum*, to highlight the diagnostic challenges posed by the marked overlap between HLH and VL, and to emphasize the importance of early recognition, appropriate diagnostic approach, and prompt targeted therapy.

## 2. Case Presentation

A 68-year-old man with no significant past medical history was admitted to our department with a one-month history of persistent fever, profound fatigue, and night sweats. One month before admission, he had been diagnosed with influenza A and received symptomatic treatment with oseltamivir. Because of persistent fever despite symptomatic therapy, empirical treatment with moxifloxacin and azithromycin for presumed community-acquired pneumonia was subsequently initiated, without clinical improvement. At the time of pneumonia diagnosis, chest radiograph showed no focal pulmonary infiltrates, and the diagnosis of pneumonia was made empirically, without further justification, at a secondary tertiary hospital.

The patient resided in a rural area of northern Greece and worked as a livestock farmer, representing an important epidemiological risk factor for zoonotic infections, including VL.

On admission, he was febrile. Physical examination revealed mild splenomegaly and palpable bilateral inguinal lymphadenopathy, while no hepatomegaly was detected. Abdominal computed tomography confirmed splenomegaly without additional abnormal findings.

Initial laboratory investigation demonstrated pancytopenia, severe hyperferritinemia (15,000 ng/mL; reference value < 306 ng/mL), hypertriglyceridemia (319 mg/dL; reference value < 150 mg/dL), and markedly elevated soluble interleukin-2 receptor (sCD25) concentrations (7619 U/mL; reference value < 2400 U/mL) (Table 1). The calculated HScore was 210, corresponding to a very high probability of reactive hemophagocytic syndrome. Given the constellation of prolonged fever, splenomegaly, and pancytopenia, an extensive diagnostic work-up was undertaken to exclude hematologic malignancy, autoimmune disease, and infectious etiologies.

The patient had no evidence of underlying immunodeficiency. Serologic testing for human immunodeficiency virus, hepatitis B virus, hepatitis C virus, Epstein–Barr virus (EBV), and cytomegalovirus (CMV) was negative. Autoimmune serological investigations and screening for tuberculosis were also unremarkable (Table 2).

Bone marrow aspiration and biopsy demonstrated activated macrophages with prominent hemophagocytosis, fulfilling one of the HLH-2004 diagnostic criteria [5]. Together with persistent fever, splenomegaly, bicytopenia, hypertriglyceridemia, marked hyperferritinemia, elevated sCD25 concentrations, and an HScore of 210, the diagnosis of secondary HLH was strongly supported [4,5]. Anti-Leishmania antibodies were detected using a commercial indirect immunofluorescence assay (IFA), whereas polymerase chain reaction (PCR) targeting Leishmania kinetoplast DNA was performed on bone marrow aspirate and identified *Leishmania infantum*, thus confirming diagnosis.

Treatment with liposomal amphotericin B was initiated according to current clinical recommendations for VL [11]. Owing to the pronounced hyperinflammatory syndrome, intravenous dexamethasone (4 mg twice daily) was concomitantly administered. The patient experienced progressive resolution of fever and marked clinical improvement during hospitalization. It has to be noted that treatment with liposomal amphotericin B was well tolerated, while no infusion reactions, nephrotoxicity, electrolyte disturbances, or other serious adverse events were documented.

**Figure 1:**
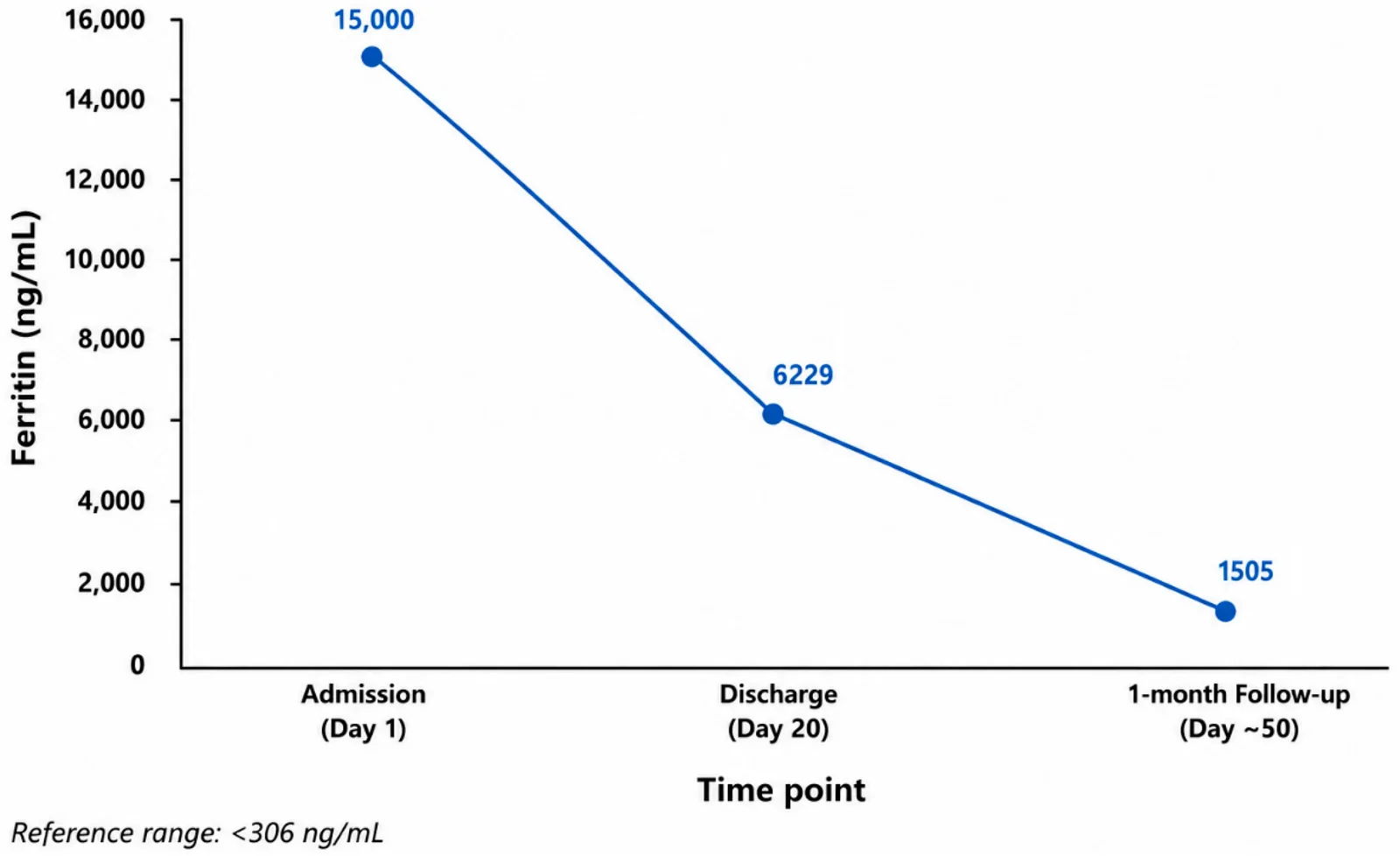
Serial ferritin measurements during hospitalization and at one-month follow-up demonstrating progressive normalization after initiation of liposomal amphotericin B and dexamethasone.

**Figure 2:**
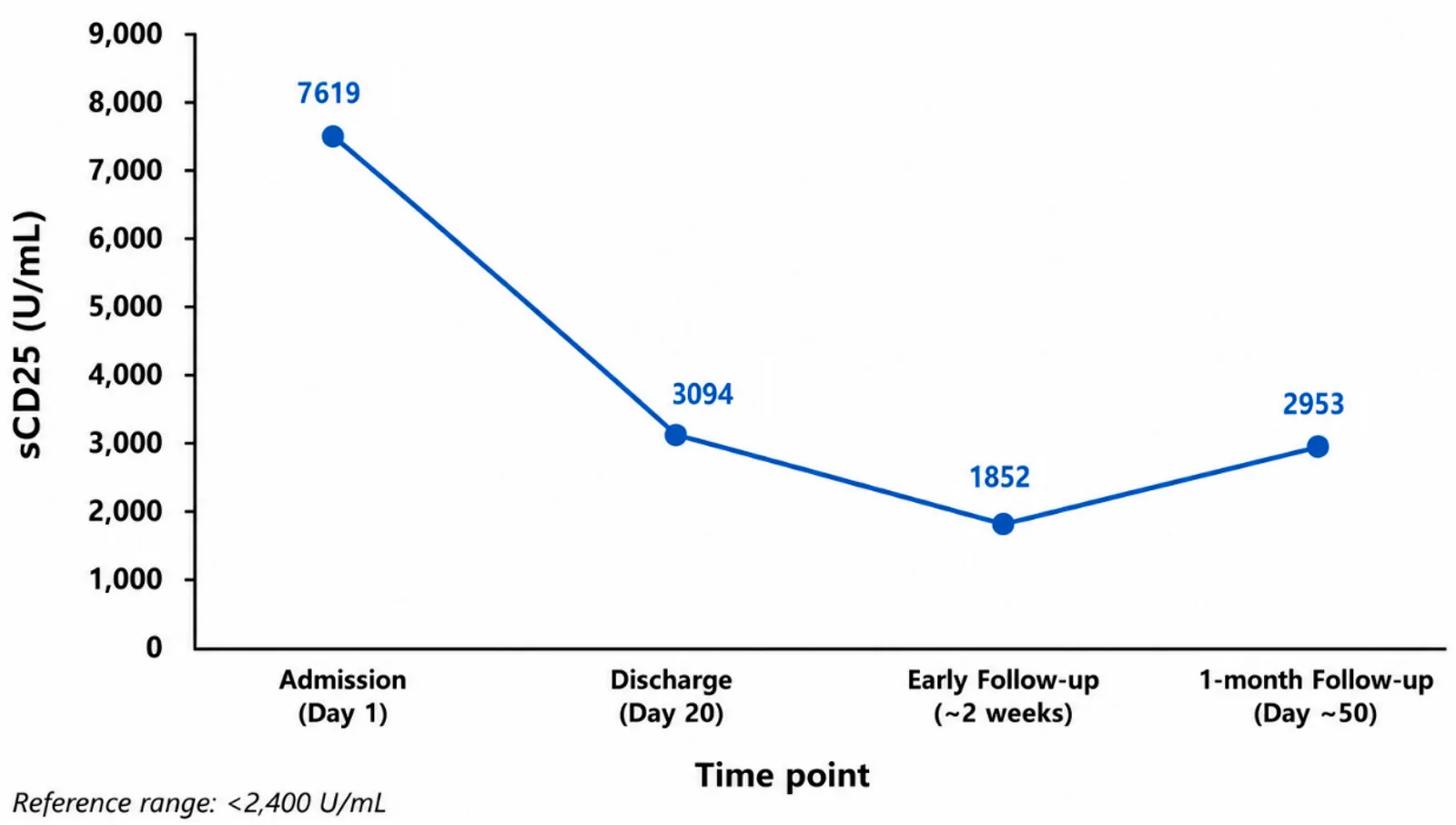
Trend of soluble interleukin-2 receptor (sCD25) levels during the disease course, illustrating resolution of the hyperinflammatory state following treatment.

Ferritin concentrations decreased from 15,000 ng/mL at admission to 6229 ng/mL at discharge (Figure 1), whereas sCD25 levels also gradually decreased (Figure 2). After a 20-day hospital stay, he was discharged in stable clinical condition with complete resolution of symptoms.

At one-month follow-up, he remained asymptomatic. Peripheral blood counts had normalized, and ferritin concentrations had further declined to 1505 ng/mL (Figure 1), consistent with sustained resolution of the hyperinflammatory syndrome.

Serial laboratory assessment after three months has demonstrated progressive resolution of the hyperinflammatory syndrome and recovery of hematologic parameters (Table 1). Ferritin levels decreased from >15,000 ng/mL at admission to 6229 ng/mL at discharge and continued to decline to 789 ng/mL at the 3-month follow-up. Similarly, C-reactive protein (CRP) and sCD25 concentrations markedly decreased over time, while hemoglobin and platelet counts normalized, consistent with sustained clinical remission (Table 1). In addition, repeat abdominal ultrasonography performed during follow-up (May 2026) demonstrated regression of both hepatomegaly and splenomegaly, consistent with the patient’s favorable clinical and laboratory response to treatment. The diagnostic investigations undertaken to exclude alternative infectious, autoimmune, and hematologic causes of HLH are summarized in Table 2.

## 3. Discussion

Although uncommon, VL is a well-recognized infectious trigger of secondary HLH. Diagnosis remains particularly challenging because both conditions share several clinical and laboratory manifestations, including prolonged fever, splenomegaly, cytopenias, hypertriglyceridemia, and marked hyperferritinemia [3,8,9]. Consequently, patients are frequently investigated for hematologic malignancies or severe systemic infections before the correct diagnosis is established, potentially delaying appropriate treatment [3].

The pathophysiological association between VL and HLH is attributed to the intracellular persistence of *Leishmania* parasites within macrophages. Continuous antigenic stimulation results in sustained activation of macrophages and cytotoxic T lymphocytes, with excessive production of pro-inflammatory cytokines including interferon-γ, tumor necrosis factor-α, and interleukin-6. The resulting cytokine storm underlies the uncontrolled immune activation characteristic of HLH and may ultimately lead to multiorgan dysfunction if left untreated [2,7].

Diagnosis of HLH in adults remains challenging, because the HLH-2004 diagnostic criteria were originally developed for pediatric patients [4,5]. Consequently, the HScore has emerged as a valuable complementary tool for estimating the probability of reactive HLH in adults. In our patient, an HScore of 210 indicated a very high probability of secondary HLH and, together with fulfillment of the HLH-2004 criteria, supported early initiation of the treatment of choice [4,5,10].

Marked hyperferritinemia remains one of the most informative laboratory abnormalities in HLH. Although ferritin concentrations exceeding 10,000 ng/mL are not pathognomonic, they strongly support the diagnosis in the appropriate clinical setting [11]. Likewise, elevated sCD25 concentrations reflect marked T-cell activation and represent an established diagnostic criterion [4,5]. In our patient, serial ferritin measurements closely paralleled clinical improvement, highlighting their usefulness for monitoring treatment response. In parallel with ferritin, serial sCD25 measurements demonstrated a marked decline following treatment, supporting its role not only as a relevant diagnostic criterion, but also as a useful biomarker of immune activation and response to applied therapeutic interventions.

Diagnosis of VL may also be difficult, particularly in patients with a low parasite burden. Although microscopic examination of bone marrow aspirates has traditionally been considered the reference diagnostic method, its sensitivity is variable. In contrast, PCR analysis provides excellent sensitivity and specificity and proved instrumental in confirming *Leishmania infantum* infection in the present case [1,6,12,13,14]. Furthermore, the patient’s occupation as a livestock farmer and residence in rural northern Greece represented important epidemiological clues that prompted investigation for zoonotic and parasitic diseases. Careful assessment of occupational and environmental exposure should therefore remain an integral component of the diagnostic evaluation in patients presenting with fever of unknown origin in endemic regions.

Management of VL-associated HLH differs from that of primary HLH or malignancy-associated HLH, because eradication of the underlying infectious trigger is the principal therapeutic objective [4,12,13]. Liposomal amphotericin B remains the treatment of choice for VL and is associated with rapid clinical improvement in most reported cases [6,13,14,15,16,17,18,19,20]. The role of adjunctive corticosteroids remains controversial. Although antiparasitic therapy alone may be sufficient in many patients, corticosteroids may be considered in selected cases with severe hyperinflammation or evolving organ dysfunction. In the present case, combined treatment with liposomal amphotericin B and dexamethasone resulted in rapid resolution of both clinical manifestations and laboratory abnormalities.

Recently published adult cases, as displayed in Table 3, consistently demonstrate that VL-associated HLH is potentially life-threatening, but highly treatable if early recognized. As also shown in Table 3, most reported patients recover after prompt initiation of liposomal amphotericin B, although some may require adjunctive immunosuppressive therapy. Compared with previously published reports, our patient shared the typical clinical triad of prolonged fever, splenomegaly, and cytopenias, but differed in that the diagnosis was established through the combined application of the HLH-2004 criteria, HScore assessment, and molecular confirmation of *L. infantum* infection by bone marrow PCR. Moreover, serial measurements of ferritin and sCD25 levels closely paralleled clinical improvement and finally recovery, underscoring their potential utility as biomarkers of treatment response, in addition to their established diagnostic role.

Although the clinical manifestations of HLH are largely determined by the underlying hyperinflammatory syndrome rather than the triggering condition, VL-associated HLH exhibits several distinctive features that may facilitate diagnosis. Similar to EBV-, CMV-, or bacterial infection-associated HLH, patients typically present with prolonged fever, splenomegaly, cytopenias, hyperferritinemia, hypertriglyceridemia, and elevated sCD25 concentrations [1,2,3]. Consequently, the underlying trigger cannot usually be identified on clinical grounds alone. However, VL-associated HLH is characterized by specific epidemiological clues and the frequent coexistence of marked splenomegaly and pancytopenia. Unlike EBV-associated HLH, which often affects younger individuals and is associated with high viral loads and aggressive disease requiring immunochemotherapy, VL-associated HLH is primarily driven by persistent intracellular parasitic infection and frequently resolves following prompt initiation of antiparasitic therapy. Likewise, HLH secondary to hematologic malignancies generally carries a poorer prognosis because successful treatment depends on controlling the underlying neoplasm in addition to suppressing hyperinflammation. In contrast, eradication of *Leishmania* infection with liposomal amphotericin B alone may be sufficient in many patients, although adjunctive corticosteroids or other immunomodulatory therapies may be required in severe cases with evolving organ dysfunction [1,2,3,8].

The present report has several limitations inherent to its single-case design. Serial measurements of inflammatory cytokines such as interleukin-6 and tumor necrosis factor-α were unavailable, and follow-up imaging was not routinely performed after clinical recovery. In addition, images of bone marrow aspirate examination were not available for inclusion in the present case report. Furthermore, conclusions regarding the benefit of adjunctive corticosteroid therapy should not be generalized, because treatment decisions were individualized. Nevertheless, the detailed diagnostic evaluation, molecular confirmation of *Leishmania infantum*, and serial laboratory follow-up provide valuable clinical insights into this uncommon presentation.

## 4. Conclusions

Secondary HLH should be considered in patients presenting with prolonged fever, splenomegaly, cytopenias, hypertriglyceridemia, and marked hyperferritinemia, particularly in regions where VL is endemic. Careful evaluation of epidemiological exposure, combined with application of the HLH-2004 criteria, HScore assessment, and molecular diagnostic techniques, facilitates early diagnosis. Prompt initiation of liposomal amphotericin B, with individualized consideration of adjunctive corticosteroid therapy, may result in complete clinical recovery and favorable long-term outcomes.

## Figures and Tables

**Table 1:** Serial laboratory parameters during hospitalization and follow-up.

Parameter	Admission (Day 0)	Discharge (Day 20)	1-Month Follow-Up (Day 50)	3-Month Follow-Up (Day 80)	Reference Range
White blood cell count (×10^3^/μL)	4.5	7.5	12.5	7.0	4.0–10.0
Hemoglobin (g/dL)	10.0	11.6	13.0	12.9	13.0–17.0
Platelet count (×10^3^/μL)	97	146	177	203	150–400
Erythrocyte sedimentation rate (ESR, mm/h)	63	–	30	29	<20
C-reactive protein (CRP, mg/L)	78.1	27.8	3.4	4.8	<5
Ferritin (ng/mL)	>15,000	6229	1505	789	<306
Soluble interleukin-2 receptor (sCD25, U/mL)	7619	3140	1940	2967	<2400

**Table 2:** Diagnostic work-up performed to exclude alternative causes of secondary HLH.

Investigation	Result	Diagnostic Implication
Complete blood count	Pancytopenia	Compatible with HLH and VL; prompted further investigation
Serum ferritin	>15,000 ng/mL	Fulfilled HLH-2004 criterion; supported hyperinflammatory syndrome
Serum triglycerides	319 mg/dL	Fulfilled HLH-2004 criterion
Soluble IL-2 receptor (sCD25)	7619 U/mL	Fulfilled HLH-2004 criterion; marker of T-cell activation
HIV serology	Negative	Excluded HIV-associated immunodeficiency
HBV serology	Negative	Excluded chronic hepatitis B infection
HCV serology	Negative	Excluded chronic hepatitis C infection
EBV serology	Negative	Excluded EBV-associated HLH
CMV serology	Negative	Excluded CMV-associated HLH
Autoimmune serology (ANA, ANCA)	Negative	No evidence of autoimmune disease or vasculitis
Tuberculosis screening (Interferon-Gamma Release Assay)	Negative	Excluded tuberculosis
Blood cultures	Negative	No evidence of bacterial bloodstream infection
Bone marrow aspiration and biopsy	Hemophagocytosis	Fulfilled HLH-2004 criterion; excluded hematologic malignancy
*Leishmania* serology	Positive	Raised suspicion for visceral leishmaniasis
Bone marrow PCR	Positive for *Leishmania infantum*	Confirmed visceral leishmaniasis as the trigger of secondary HLH

**Table 3:** Representative published adult cases of secondary hemophagocytic lymphohistiocytosis associated with visceral leishmaniasis due to *Leishmania infantum*. Abbreviations: M: male; F: female.

Study	Year	Age/Sex	Country	*Leishmania* Species	Treatment	Outcome
Rajagopala et al. [8]	2008	Adult	India	Visceral *Leishmania* spp.	Liposomal amphotericin B ± corticosteroids	Recovery
Cançado et al. [16]	2013	79/M	Brazil	*L. infantum* (syn. *L. chagasi*)	Amphotericin B	Recovery
Watkins et al. [17]	2014	73/M	Spain (traveler)	*L. infantum*	Liposomal amphotericin B	Recovery
Neycheva et al. [18]	2021	40/F	Bulgaria	*L. infantum*	Cyclosporine, corticosteroids, followed by liposomal amphotericin B	Recovery
Qin et al. [6]	2022	Five adult cases	China	Visceral *Leishmania* spp.	Sodium stibogluconate or liposomal amphotericin B	Most patients recovered
Joudeh et al. [19]	2023	20/M	Qatar	*L. infantum*	Liposomal amphotericin B	Recovery
Bougiouklou et al. [20]	2026	60/M 72/M	Greece	*L. infantum*	Liposomal amphotericin B; one patient additionally received intravenous immunoglobulin	Recovery (both)

## Data Availability

The data that support the findings of this study are available upon request from the corresponding author. The data are not publicly available due to privacy or ethical restrictions.
